# Barriers and enablers of pelvic floor rehabilitation behaviours in pregnant women with stress urinary incontinence: a qualitative analysis using the theoretical domains framework

**DOI:** 10.1186/s12884-023-05633-2

**Published:** 2023-04-28

**Authors:** Ping Xu, Ying Jin, Pingping Guo, Xuefen Xu, Xiaojuan Wang, Wei Zhang, Minna Mao, Suwen Feng

**Affiliations:** 1 Zhejiang University School of Medicine, Hangzhou, Zhejiang China; 2 Women’s Hospital, Zhejiang University School of Medicine, No.1 Xue Shi Road, Hangzhou, Zhejiang Province 310006 People’s Republic of China

**Keywords:** Pregnant women, Urinary incontinence, Theoretical domains framework, Pelvic floor muscle exercises, Lifestyle interventions

## Abstract

**Background:**

Stress urinary incontinence during pregnancy is closely related to the occurrence of postpartum and long-term urinary incontinence. Early pelvic floor management is of great significance in promoting the recovery of pelvic floor tissues in pregnant women. However, effective management of urinary incontinence is far from achievable owing to the low adherence of pregnant women in partaking in pelvic floor rehabilitation. As a comprehensive framework for behavioural theory, the Theoretical Domain Framework allows for comprehensive identification of behavioural determinants. Using Theoretical Domain Framework, this study aimed to identify barriers and enablers of pelvic floor rehabilitation behaviours in pregnant women with stress urinary incontinence.

**Methods:**

A descriptive, qualitative design was used in this study. Face-to-face semi-structured interviews were conducted with pregnant women with stress urinary incontinence based on the Theoretical Domain Framework. The data were analysed using a combination of inductive and deductive methods.

**Results:**

Twenty pregnant women with stress urinary incontinence were interviewed. Seven themes were summarised and used to explain the pelvic floor rehabilitation behaviours of pregnant women with stress urinary incontinence. The seven themes were (1) individual knowledge and experience of pelvic floor management, (2) judgments about expected outcomes, (3) interactions of interpersonal situations, (4) environment, resources, and decision-making processes, (5) personal goal-setting and efforts towards behaviour change, (6) emotional influences on decision-making, and (7) personal characteristics. Besides the "Optimism" domain, 13 of the 14 Theoretical Domains Framework domains were found to influence pregnant patients' pelvic floor rehabilitation behaviours after deductive mapping of themes to the Theoretical Domains Framework. In addition, the inductive analysis generated a theme of personal characteristics that did not map to any of the Theoretical Domains Framework domains.

**Conclusions:**

The pelvic floor rehabilitation behaviours of pregnant women with stress urinary incontinence are complex and are affected by many factors. The findings confirm the need for multiple interventions to support pelvic floor management in pregnant women with stress urinary incontinence, focusing on enhancing knowledge and skills in pelvic floor care and using appropriate behaviour change techniques (such as prompts) to provide a supportive environment.

**Supplementary Information:**

The online version contains supplementary material available at 10.1186/s12884-023-05633-2.

## Background

Urinary incontinence, one of the most common chronic diseases that endanger women health, has become an important public health and social problem. Urinary incontinence frequently prevents patients from engaging in social interactions and employment-related tasks, interferes with sexual functions, leads to embarrassment, anxiety, depression, and even social isolation, subsequently reducing the quality of life and increasing the socioeconomic burden [1–3]. Pregnancy and delivery are important risk factors for the development of urinary incontinence in women [4]. According to published data, the prevalence of urinary incontinence in pregnant women ranges from 32 to 64% [5]. Stress urinary incontinence is the most common type of urinary incontinence that occurs during pregnancy. Several studies have shown that stress urinary incontinence during pregnancy is closely associated with the occurrence of postpartum and long-term urinary incontinence. A study by Dolan et al. found that women who developed stress urinary incontinence in their first pregnancy had a two-fold higher risk of recurrent urinary incontinence within 15 years postpartum than women who did not experience urinary incontinence during their pregnancy [6]. The Schytt study showed that pregnant women with stress urinary incontinence had an increased risk of medium-to long-term urinary incontinence postpartum or later in life [7]. Therefore, early pelvic floor management is of great significance in promoting the recovery of pelvic floor tissues in pregnant women and reducing the incidence of postpartum and long-term urinary incontinence.

International clinical guidelines recommend pelvic floor muscle exercises and lifestyle interventions (e.g. weight loss, dietary changes, reduced caffeine intake, controlled fluid intake, and smoking cessation, etc.) as first-line treatment for women with urinary incontinence [8–11]. There is growing evidence that pelvic floor muscle exercises and lifestyle behavioural changes are safe and feasible treatment modalities to promote pelvic floor rehabilitation during pregnancy and can reduce symptom burden, decrease the incidence of long-term urinary incontinence, and optimise health outcomes [12, 13].

However, despite the existence of clinical guidelines based on rigorously- researched evidence for the treatment of urinary incontinence, there is still a gap between the clinical management behaviours recommended by these guidelines and the actual self-management behaviours of patients. Studies have reported that approximately 64% of patients are able to adhere to pelvic floor muscle exercises in the short term, while only 23% of patients are able to adhere to pelvic floor muscle exercises in the long term [14]. Two cross-sectional studies found that the participation rates of pregnant women in pelvic floor muscle exercises were only 3% and 11%, respectively [15, 16]. In addition, several pregnant women have a low level of knowledge about urinary incontinence and are yet to establish a good lifestyle in their daily lives. Effective prevention and treatment of urinary incontinence are far from achievable owing to the low adherence of pregnant women with pelvic floor rehabilitation behaviours (exercise, changing unhealthy lifestyle habits, etc.).

Improving adherence to pelvic floor rehabilitation behaviours in pregnant women is crucial for the control and treatment of urinary incontinence. To promote behavioural changes in pregnant women with urinary incontinence, it is important to first understand the barriers and enablers of pelvic floor rehabilitation behaviours. Recent studies have reported that the most common reasons that prevented urinary incontinence patients from performing pelvic floor muscle exercises included forgetting to exercise, lack of time, and boredom with the exercise [17–19]. However, these studies on the adherence to pelvic floor rehabilitation behaviours have mainly been conducted in postpartum and general urinary incontinence patients; therefore, evidence on adherence to pelvic floor rehabilitation behaviours in pregnant patients remains limited. Furthermore, it is important to note that current investigations have focused on quantitative studies and have often failed to adequately explore the reasons behind behavioural changes in pelvic floor rehabilitation in pregnant women with stress urinary incontinence.

The Medical Research Council’s guidance on the development of complex interventions advocates the application of theory to identify the mechanisms of behavioural change [20]. The Theoretical Domains Framework (TDF), developed and validated by Michie and other researchers, integrates 33 psychological theories and 128 theoretical structures and is an essential integrative framework for the behaviour change theory [21, 22]. The TDF can be used to explore the barriers and facilitators of complex healthcare behaviours, thereby providing information for intervention design. Specifically, the TDF includes 14 theoretical domains representing behavioural determinants, including: "Knowledge," "Skills," "Memory, attention and decision processes," "Behavioural regulation," "Environmental context and resources," "Social influences," "Social/professional role and identity," "Beliefs about capabilities," "Beliefs about consequences," "Optimism," "Intentions," "Goals," "Reinforcement" and "Emotions" [23]. Potentially modifiable factors in the 14 structural domains can be linked to interventional function and behavioural change techniques to guide intervention design [24]. The TDF has proven to be useful for investigating the barriers and enablers of exercise and lifestyle management in a variety of groups [25–29]. However, to date, no study has applied TDF to explore the factors influencing adherence with pelvic floor rehabilitation behaviours in pregnant patients with urinary incontinence. Therefore, the objectives of this qualitative study were to identify barriers and facilitators of the implementation of pelvic floor rehabilitation behaviours in pregnant women with stress urinary incontinence using a TDF-based semi-structured interview to lay a foundation for designing effective behaviour change interventions and promoting adherence of pelvic floor management behaviours in pregnant women with urinary incontinence.

## Methods

### Study design

This study used a descriptive, qualitative design. Descriptive qualitative methodology enables researchers to explore the perspectives and experiences of participants [30]. Semi-structured one-to-one interviews were conducted to explore the experience of pregnant women with stress urinary incontinence in dealing with urinary leakage and gain a detailed understanding of the factors that facilitate or hinder patients from performing pelvic floor rehabilitation behaviours. The TDF was used to guide data collection and analysis. This study was reported in accordance with the COREQ statement (Additional file 1) [31].

### Setting

This study was conducted at a large Grade A tertiary hospital in Hangzhou, China, from December 2021 to July 2022. The hospital has departments of gynaecology, obstetrics, and women's health-care, which mainly provide services such as pregnancy care, postpartum rehabilitation, and gynaecological oncology treatment to patients. This hospital has 1,120 beds and 1.6 million annual outpatient visits and is a Grade A tertiary obstetrics and gynaecology hospital with great influence in eastern China and the country as a whole.

### Participants

Face-to-face recruitment and screening of pregnant women was conducted by head nurses; the screening questions are listed in Table 1. The head nurses verbally invited all pregnant women identified as having stress urinary incontinence to discuss their experiences managing urinary incontinence with the researcher. The head nurses gave the participants verbal information about the study and asked for permission to be contacted by the researcher. Our researchers obtained a list of potential participants (pregnant women with stress urinary incontinence) through the head nurses. We adopted a sampling strategy that combined maximum variation sampling with standard sampling to select eligible participants. Initially, we used standard sampling methods to select the respondents. Detailed information on the inclusion and exclusion criteria for participants is presented in Table 2. The researchers analysed the inclusion and exclusion criteria, and this process identified that 53 women were eligible to participate in the study. Subsequently, maximum variation sampling was used to recruit patients for interviews. Potential participants were purposefully sampled by characteristics including age, gestational age, education, and socioeconomic status to ensure the diversity and representation of participants and collect a wealth of information through the study questions. After patients gave their consent, researchers contacted potential participants by phone. Twenty-three eligible patients were invited to participate in the study, but three declined the invitation. Busy schedules were the main reason for participants declining the invitation.

**Table 1:** Screening questions

Question and Answer (Q&A) Session	
1. First, ask the pregnant woman if she experiences urinary incontinence	
Do you have urine leakage?	• Instruct the pregnant woman to answer "never", "sometimes", or "always" ➢ When the pregnant woman answers "always" or "sometimes", it means the pregnant woman experiences urinary incontinence. So, continuing with the next question ➢ When the patient answers "never", it means there is no urinary incontinence and the Q&A is over
2. Next, ask the patient about the type of urinary incontinence	
(1) Do you leak urine when coughing, sneezing, laughing, lifting heavy objects, or exercising?	• Instruct the patient to answer "always", "sometimes" or "never" ➢ When the pregnant woman answers "always" or "sometimes", it means that the pregnant woman experiences stress urinary incontinence. So, moving on to the next question ➢ When the patient answers "never", it means the absence of stress urinary incontinence and then ends the Q&A
(2) Do you leak urine on your way to the toilet?	• Instruct the patient to answer "always", "sometimes" or "never" ➢ When the patient answers "always" or "sometimes", it means that the patient experiences urge urinary incontinence. If stress urinary incontinence and urge urinary incontinence are both present, the patient experiences mixed urinary incontinence. The patient was excluded from this study ➢ When the patient answers "never", it means the absence of urge urinary incontinence, and then the patient is invited to participate in the study

**Table 2:** Inclusion and exclusion criteria of the study participants

Inclusion criteria	Exclusion criteria
Age ≥ 20 years old	Women with prior surgery for incontinence or pelvic organ prolapse
Symptoms of stress urinary incontinence have occurred at least once in the last month	Women with pregnancy-related complications, such as hypertension, heart disease, diabetes, etc
Women in the second and third trimesters (≥ 13 weeks of gestation)	Women suffer from cognitive impairment or mental illness
Volunteer to participate in the study and share their disease management experiences	Women who are unable to speak Mandarin

The study sample size was based on data saturation, and we followed the principles stated by Francis et al. for determining saturation in theory-based qualitative research [32]. After analysing at least ten interviews, data collection was stopped when no new codes or themes were identified from three more consecutive interviews.

### Ethics

This study was approved by the ethics committee of the Women’s Hospital, Zhejiang University School of Medicine (no.: IRB-20210342-R). All participants provided written informed consent before the interviews. All participants were informed that participation in the study was voluntary and that they could choose to withdraw from the study at any time or refuse to answer any questions without providing reasons, and this would not have any detrimental effects on their care and treatment. Any personal information revealed during the interviews was deleted to protect patient privacy, and participants were assigned numbers instead. Only study members had access to the data, and all data related to this study were securely stored on a password**-**protected computer.

### Data collection

A semi-structured interview outline was developed based on the TDF (Additional file 2). Open-ended questions and questions based on each of the 14 TDF domains were included in the interview. One to three questions were developed to map each of the 14 TDF domains. Prompts were prepared for the interviewees to explain and further explore the corresponding management behaviours. Based on the interview outline, the first author conducted two pilot interviews with participants who met the research criteria. Because no additional modifications were required, the pilot data were included in the main analysis.

Before the interviews, the interviewer explained the purpose and significance of the study and obtained written informed consent from the interviewees. Each interview was conducted face-to-face with the patient by the first author in a conference room of the hospital, and all interviews were conducted in Mandarin Chinese. Each interview was recorded with the permission of the participants using handheld recording equipment to facilitate transcription. Only the participants and the researcher were present at the interview, no repeat interviews were conducted with the participants, and no transcripts were sent back to the participants for correction. Field notes were taken by the interviewer during the interviews to record the emotional changes and body language of the interviewees. Before the interview, all participants self-completed the relevant demographic and disease-related information surveys, such as age, residence, education level, gestational week, and the International Consultation on Incontinence Questionnaire-Short Form (ICIQ-SF) score. The ICIQ-SF was used to assess the severity of urinary incontinence [33]. The severity of urinary incontinence was classified according to the score as follows: mild (symptom score less than or equal to 7), moderate (symptom score range 8–13), or severe (symptom score range 14–21). It has been demonstrated that the Chinese version of the questionnaire has high validity and reliability [34].

### Data analysis

All quantitative analyses were performed using SPSS version 25.0. The demographic characteristics of the participants were reported using descriptive statistics. Continuous variables were expressed as mean ± standard deviation (SD) or median and interquartile range (IQR), and the results of the counting data were expressed as frequency numbers and percentages (%). Within 24 h of the interview, the researcher transcribed the recording verbatim, and the transcripts were checked for accuracy by another researcher. Anonymous interview data were imported into the NViVo 12 for data management and analysis. Since the themes obtained by simple deductive analysis can lose important information because they are limited to predefined TDF domains [35], this study adopted a data analysis method combining induction and deduction. The data were analysed in two stages: (1) theme analysis [36], which was used to identify the hindering and promoting factors of pelvic floor rehabilitation activity, and (2) deductive mapping of all themes to the relevant TDF domains.

#### Phase I: thematic analysis

The thematic analysis was guided by a research question: what factors influence decision-making related to women's pelvic floor rehabilitation behaviours? The researchers initially familiarised themselves with the data by repeatedly reviewing the audio recordings, interview transcripts, and field notes. Then, all data were coded inductively based on the interpretation of the data related to the research question. Data were coded primarily at the semantic level, but latent (implicit) interpretations were also considered. All transcripts were analysed by one author and independently reviewed by another author to ensure the reliability of the coding. Disagreements were resolved by a third researcher. Codes were grouped and merged according to their inherent relationships and subsequently reviewed and defined to form themes and sub-themes. Codes with similar meanings and associations were merged into sub-themes. The sub-themes were refined and collated to develop themes based on relevance, content, and meaning. Themes captured meaningful patterns of data related to the research question. By rereading the data several times and checking that the themes matched the coding, the final themes were determined through discussion and consensus among the researchers.

#### Phase 2: deductive mapping of all subthemes or themes to the relevant TDF domains

At the level of subthemes or themes, mapping was performed. Subthemes or themes were deductively mapped to the relevant domains of the TDF to identify personal and environmental factors associated with behavioural changes in pelvic floor rehabilitation in pregnant women with stress urinary incontinence. The researchers referenced the original articles related to TDF for guidance on how to map themes to each domain. If the subthemes were relevant to more than one TDF domain, they were merged into the most relevant domain. If there were any "empty" domains, the researchers would reexamine the data to determine if anything pertinent had been omitted from the coding process. To facilitate future intervention development in pelvic floor rehabilitation, the researchers further categorized each subtheme into barriers to and enablers of pelvic floor rehabilitation behaviours. Any disagreements were resolved through discussion among the researchers.

### Reflection

This study is part of the first author's PhD project, which aimed to improve adherence with pelvic floor rehabilitation behaviours in pregnant women with stress urinary incontinence. The first author, who was a PhD student in nursing science at the time of the study and had a master’s degree in nursing, had no previous relationships with the participants. Simultaneously, the first author studied the behaviour change theory, completed qualitative research-related courses prior to the research, and had extensive professional knowledge in pelvic floor care. All other members of the research team had a background in pelvic floor rehabilitation or maternal care.

## Results

### Participant characteristics

Overall, a total of 20 pregnant women with stress urinary incontinence eventually completed face-to-face interviews for the study, and none of the patients dropped out. No repeat interviews were conducted and data saturation occurred at the 20th interview. The duration of each interview ranged from 19 to 52 min, with an average duration of (30.0 ± 8.7) minutes. The average age of study participants was (30.9 ± 4.2) years old, ranging from 25 to 40 years old. The median gestational age at the time of the interview was 36 weeks, ranging from 20 to 40 weeks. The ICI-Q-SF score of the participants ranged from 4 to 16, with an average score of (8.5 ± 3.9). In addition, in terms of urinary incontinence severity, 40% of participants had mild urinary incontinence, 50% had moderate urinary incontinence, and 10% had severe urinary incontinence. The detailed characteristics of the participants are shown in Table 3.

**Table 3:** Participant characteristics (N = 20)

Number	Age	Residence	Employment status	Education level	Method of payment of medical costs	Household income per capita monthly (RMB)	Pre-pregnancy BMI	Gestational week	Pregnancy category	ICI-Q-SF/The severity of urinary incontinence	The interview durations (min)
P01	33	City	on-the-job	University	Medicare	10,000–15,000	Overweight	20	Multigravida	13/Moderate	21
P02	28	City	on-the-job	University	Medicare	5001–10,000	Normal	37	Primigravida	13/Moderate	29
P03	33	City	on-the-job	Masters and above	Self-pay	15,000–20,000	Normal	40	Multigravida	4/Mild	32
P04	34	City	off-the-job	University	Medicare	> 20,000	Normal	39	Primigravida	9/Moderate	40
P05	35	City	on-the-job	Masters and above	Medicare	15,000–20,000	Normal	38	Multigravida	4/Mild	29
P06	26	City	on-the-job	High school	Medicare	5001–10,000	Normal	39	Primigravida	4/Mild	38
P07	39	City	on-the-job	University	Medicare	5001–10,000	Normal	40	Multigravida	9/Moderate	44
P08	30	City	on-the-job	University	Medicare	5001–10,000	Normal	36	Primigravida	4/Mild	22
P09	32	City	on-the-job	Masters and above	Medicare	10,000–15,000	Underweight	38	Primigravida	12/Moderate	32
P10	27	City	on-the-job	Junior college	Medicare	10,000–15,000	Normal	31	Multigravida	8/Moderate	27
P11	28	City	unemployed	Junior college	NCMS	5001–10,000	Normal	30	Primigravida	15/Severe	22
P12	27	Rural	on-the-job	University	Medicare	5001–10,000	Normal	40	Primigravida	10/Moderate	22
P13	34	Rural	on-the-job	Junior college	Medicare	5001–10,000	Normal	30	Primigravida	7/Mild	19
P14	40	City	on-the-job	University	Medicare	5001–10,000	Normal	36	Multigravida	5/Mild	31
P15	25	City	on-the-job	Junior high school	Medicare	5001–10,000	Normal	39	Primigravida	8/Moderate	52
P16	25	City	on-the-job	Junior college	Medicare	15,000–20,000	Normal	27	Primigravida	8/Moderate	25
P17	31	City	on-the-job	Junior college	Medicare	15,000–20,000	Normal	26	Primigravida	16/Severe	39
P18	29	City	off-the-job	University	Medicare	5001–10,000	Normal	25	Primigravida	4/Mild	23
P19	30	City	on-the-job	University	Medicare	> 20,000	Normal	26	Primigravida	5/Mild	26
P20	32	City	on-the-job	Junior college	Medicare	10,000–15,000	Normal	32	Primigravida	11/Moderate	27

*Abbreviations*: *NCMS* New cooperative medical scheme; International Consultation on Incontinence Questionnaire-Short Form (ICIQ-UI-SF). BMI: underweight (BMI < 18.5); normal weight: (18.5 ≤ BMI < 24), overweight: (24 ≤ BMI < 28); obese: (BMI ≥ 28)

### Barriers and enablers of pelvic floor rehabilitation behaviours in pregnant women with urinary incontinence

Seven themes and corresponding sub-themes were summarised from the data, which were then deductively mapped to the 13 TDF domains. The results are presented in Table 4. The seven themes were (1) individual knowledge and experience of pelvic floor management, (2) judgments about expected outcomes, (3) interactions of interpersonal situations, (4) environment, resources, and decision-making processes, (5) personal goal-setting and efforts towards behaviour change, (6) emotional influences on decision-making, and (7) personal characteristics. Mapped to the 13 TDF domains are "Knowledge," "Skills," "Memory, attention and decision processes," "Behavioural regulation," "Environmental context and resources," "Social influences," "Social/professional role and identity," "Beliefs about capabilities," "Beliefs about consequences," "Intentions," "Goals," "Reinforcement" and "Emotions." However, the themes generated from the inductive analysis did not map to the "Optimism" domain of TDF. In addition, the inductive analysis generated a theme of personal characteristics that did not map to any of the Theoretical Domains Framework domains.

**Table 4:** Summary of barriers and enablers to performing pelvic floor rehabilitation behaviours mapped to the Theoretical Domains Framework domains

Theme	Sub-theme	Barrier/enabler	TDF domain	The representative interview quotation
Individual knowledge and experience of pelvic floor management	Knowledge of urinary incontinence	Enabler	Knowledge	So, is it mainly because my ability to control my pelvic floor muscles is weak? Control of that piece of muscle, right? The reason for pelvic floor muscle exercises is that the pelvic floor muscles are flabby and cannot provide adequate support. For example, the two muscles in the isthmus may be relatively flabby, and some pelvic structures may not be supported adequately. Muscle… are the muscle fibres not good? Right? [P05]
	Incorrect or very limited knowledge on pelvic floor management	Barrier	Knowledge	I do not know the classification of urinary incontinence. I have not experienced urinary incontinence. I only experienced urine leakage when I had a cough or cold. [P11] I have not paid much attention to lifestyle. I really do not understand the diet, and I do not understand what effect it will have on the pelvic floor muscles or this kind of anti-urinary incontinence. [P03]
	Good skills in pelvic floor management	Enabler	Skills	Kegel training… tightening (pelvic floor muscles), tightening, and releasing. [P11]
	Lack of skills in pelvic floor management	Barrier	Skills	I preferably need a training course or some kind of guidance. I am afraid of doing it the wrong way. [P04] I definitely need training to raise my awareness of pelvic floor management. I know only little about this. There are courses in Hospital A, are there not? However, although I have learned a little, I feel it is superficial and not very deep. [P09]
	Have the confidence to control the disease	Enabler	Beliefs about capabilities	Where are those muscles? I know. He taught me (referring to the coach). Is it Kegel? I know it so well that I can help you in writing this paper, ha-ha. [P06]
	Uncertainty about one's own abilities	Barrier	Beliefs about capabilities	It is easy to do this (pelvic floor management), but I may not be able to adhere to it. This is mainly a matter of persistence. It is just like we do with weight loss exercise: think of it and then jump the rope twice. However, it is difficult to adhere to. [P12] I do not know if I can stick to pelvic floor management. Like losing weight, one should just be willing to exercise for a period of time. Pelvic floor management it is good, is it not, ah? Eating less and sleeping more is also good; it is the same thing; you cannot all be sticking to it and have a good habit every day. [P05]
Judgments about expected outcomes	Perceived benefits of pelvic floor management and the consequences of non-adherence	Enabler	Beliefs about consequences	It is better to exercise than not to exercise, and you are definitely better off tighter than flabby. I think during my pregnancy I might have leaked a bit, as we all do. If I do not exercise after giving birth or if I have this kind of frequent urination or urinary incontinence, it is not a good feeling. It is different from other people, and it is abnormal. Thus, it would be better to return to a relatively normal level. [P18] There are some negative consequences of urinary incontinence, such as muscle relaxation and urine leakage, when you get older, which can have an impact on your life. If you do not manage it well, you are going to feel like you are causing a lot of trouble. Each time you sneeze, you would have to change your underwear or would need protective pads or something, and you have to find a place for such sanitary purposes. Do you have to take a bunch of things with you when you go out? [P10] I do not sit, stand, or lie down for long periods of time because it causes constipation and tailbone pain, so I subconsciously walk a little after sitting for a long time. [P18]
	Doubts about the value of pelvic floor management	Barrier	Beliefs about consequences	I know that recovery varies from person to person. Some people have actually done so and may not have recovered very well. [P12]
Interactions of interpersonal situations	Positive impacts on socio-cultural	Enabler	Social influences	(Kegel training) post-95 s all know I think. [P06]
	Negative influences of socio-cultural	Barrier	Social influences	I asked people around me about my urine leakage, and I have also asked those who have given birth to children around me. They said it was normal, and they said that you could repair it after you have given birth, and they all answered in this way. I can say that this is completely blank because I have also come to this conclusion from living examples around me. When my friends around me gave birth to their children, none of them had this awareness. In other words, the awareness of pelvic floor management during pregnancy should be relatively blank. [P17]
	Accessibility to social support	Enabler	Social influences	(1) Information and advice provided by the medical team Unless you are seeing a doctor whose guidance is important. [P05] (2) Support from family members I do not know if my family and husband will support me because they cannot feel the pain. First, if this is not particularly serious, it may not have a great impact on my life, making it even more impossible for them to understand my confusion. If there is help and reminders from family members, it may be very good. If my family feels that it does not matter because they cannot understand me either, I think would be a bit difficult to stick with this if I am completely on my own. [P14] (3) Peer support Like in any other sport, if you feel like you are the only person participating in this activity and you have no partner, you might feel confident at the beginning, but after a while, you might start to feel tired, frustrated, or for a variety of other reasons, you may give up slowly. If there was a group of friends at that time, they might say, for example, "Eh, did you exercise today" or something like that. There may be mutual supervision. [P14] (4) Network support from local communities and other groups Some of my colleagues had babies, and after their second child, they said that the leakage was more severe. In fact, this unit or one of your commonweal organizations may promote this Kegel exercise for pelvic floor muscle rehabilitation. I can the integrate into a certain exercise group or a yoga group, and then you may combine this kind of commonweal promotion with this kind of union of a certain unit so we can all form an atmosphere where we can exercise together, right? It is a very good thing to spend time exercising collectively during working hours. Nowadays, many trade unions also organise activities, but not specifically for the pelvic floor. I think it would be ideal if you were to promote it from an advocacy point of view, that is, an initiative, and then let us cooperate. [P07]
Environment, resources, and decision-making processes	Objective constraints lead to forgetfulness	Barrier	Memory, attention and decision processes	When I am busy at work, something like raising a baby, or something else, will distract me and I will not think about it at all. [P10] There will be instances when I forget to exercise. If I choose to exercise every day of the week, I may miss two or three days. [P06] It is truly necessary to focus on this aspect of pelvic floor management and provide time away from home for exercise. I am not free when I get home. Do you understand? [P07] An alarm clock will help remind me of my own pelvic floor management. [P20]
	The high accessibility of information	Enabler	Environmental context and resources	Online resources are available. During this time, you might find this kind of information on Tiktok or whatever. Xiaohongshu also contains this information. [P04]
	Variable quality of information	Barrier	Environmental context and resources	There are several information on the internet. Some of these information may be good knowledge, while others may be incorrect. [P14]
	Limited comprehensibility of information	Barrier	Environmental context and resources	I prefer to obtain information that is simple and straightforward, that can be understood at once. I might not be able to understand it if it is boring and tedious. [P02]
Personal goal-setting and efforts towards behaviour change	Perceived poor physical condition increases individual willingness to manage	Enabler	Intentions	The possibility of sticking to pelvic floor management for me was 100%. I definitely want to improve myself. I am sure I will stick to it. If I know I have a problem or something, I am sure I will be able to change it and achieve the best possible state. [P04]
	Perceived good physical condition weakens individual management intention	Barrier	Intentions	As before, after I gave birth to my first child, I found out that I had incontinence, so I might have looked online to find out why it was happening. However, because it did not have a big impact on my life, I did not say I had to perform pelvic floor management to improve it (laughs). [P14]
	Low priority	Barrier	Goals	I think brushing your teeth is necessary every day, but if you do not brush your teeth, for example, you will have tooth decay, and there will be many subsequent problems later. I feel that (pelvic floor management) may not be as important. [P19]
	Self-responsibility	Enabler	Social / professional role and identity	I will manage this myself. Because you have this problem, you must solve it. I do not want to have an unpleasant disease because I am a traditional Chinese medicine practitioner myself, and I want to be healthy because there are some things—the same as traditional Chinese medicine, I will give you the best technology and best therapies. If you do not exercise or self-manage, it will not work. I think this thing is still on your own. [P15]
	Behaviour change strategies	Enabler	Behavioural regulation	I would probably perform pelvic floor muscle exercises before going to bed or at a fixed time anyway. As such, I think it may not have to be a special method, and it may be possible to practice while lying on the bed. Maybe I could perform the exercises before going to bed. [P05]
	Self-motivation	Enabler	Reinforcement	Buy yourself a bag. [P06]
Emotional influences on decision-making	Safety in pelvic floor management	Enabler	Emotions	There seems to be nothing special about this treatment. This is a type of muscle exercise. I did not hear anything about side effects, so I do not have any concerns. [P03]
	The emotional burden of urinary incontinence	Barrier	Emotions	I do not exercise if I am in a bad mood. [P06] I am more annoyed and worried. If I sneeze later, will there be (leakage of urine)? [P10]
Personal characteristics	Positive impacts on personal characteristics	Enabler	Not applicable	Be rational about things, such as urinary incontinence, as it often occurs. This is because pregnancy is stressful, right? Suddenly, the belly gets bigger and the body has urinary incontinence. [P06] I will manage this myself. I do not like to say that I just like to do it, and then have a problem solving it. I am this kind of person. [P15]
	Negative influence of personal characteristics	Barrier	Not applicable	What gets in the way of pelvic floor management is laziness. Lazy people do not want to move. [P04]

#### Individual knowledge and experience of pelvic floor management

##### Knowledge

Knowledge is both an enabler of and a barrier to pelvic floor rehabilitation in pregnant women with stress urinary incontinence. The results of the interviews revealed that the level of knowledge of the respondents on pelvic floor management varied. Several participants mentioned their knowledge on urinary incontinence. Participants were able to provide examples of people who may be at risk of urinary incontinence. Additionally, the participants discussed the reasons for pelvic floor management as well as the methods of lifestyle modifications.

*Regarding the scientific diet, I cannot have this kind of behaviour. For example, I often drink most of my water in one gulp. I may choose to hold the water in my mouth and drink slowly, and may drink a cup of water within one hour. This facilitates my metabolism and ensures that my urine is not concentrated. [P07]*

However, many patients still have incorrect or very limited knowledge on urinary incontinence and its management. During the interviews, several participants reported that it was their first time of hearing about pelvic floor management during pregnancy. A few participants claimed that pelvic floor management during pregnancy is not as urgent or necessary as postpartum pelvic floor care. Furthermore, interviews with patients showed that most patients were unable to clarify the specific content of lifestyle management and did not have a good understanding of the specific methods of pelvic floor muscle exercises. This lack of knowledge has a significant negative impact on pelvic floor rehabilitation behaviours.

*I may not have performed pelvic floor muscle exercises during pregnancy. Because of the information I received, I felt as if this was something I would do in the future after I had the baby. Therefore, I may not have thought about this before, and I think it is a question to be considered later. [P08]*

##### Skills

A few participants reported of having developed good pelvic floor management skills when asked how to control and manage urinary incontinence.

*When you want to sneeze and feel it, you have to deliberately control the pelvic floor muscles on your own. [P02]*

However, majority of the participants reported that they did not receive any formal training on pelvic floor management, their knowledge was mostly obtained through online access, which did not always ensure correct practices in pelvic floor management due to a lack of professional training. Moreover, they emphasised the need for professional training in pelvic floor management to gain in-depth information on pelvic floor management, such as identifying pelvic floor muscles, taking correct diet, drinking, and exercising.

*Pelvic floor muscles… could this be… the hips? The sides of the hips? [P12]*

##### Beliefs about capabilities

Participants varied substantially in their self-perceived confidence in pelvic floor management; therefore, beliefs about capabilities could be barriers or facilitators. A few participants made it clear that they felt very confident in their ability to manage the pelvic floor muscles, and we found that patients who expressed this confidence had previously received the necessary education and training.

Most participants reflected that pelvic floor management was not difficult and that the main challenge was adherence to management practices. The participants’ feedback reflected that the frequency of individual behaviours tended to decrease dynamically over time, and they highlighted the uncertainty regarding long-term adherence to pelvic floor management.

*In fact, performing pelvic floor muscle exercises are relatively simple. However, the main thing is to persist. I think, in the beginning, If I can start performing the exercises, I might be able to do it. After a period of time, I might not be able to do it multiple times in day, unless I naturally develop the habit of doing it, like brushing my teeth and washing my face in the morning. However, if I do not have that habit, I think I might just think of it and do it. [P14]*

#### Judgments about expected outcomes

##### Beliefs about consequences

Understanding the benefits of pelvic floor management and consequences of nonadherence is a key factor in improving adherence to pelvic floor rehabilitation behaviours among participants. Nearly all participants (who were aware of the distress caused by urinary incontinence) firmly believed in the potential benefits of pelvic floor management and expressed hope that through pelvic floor management, they could control symptoms as soon as possible, change their disease status, improve pelvic floor muscle strength, and avoid the health risks associated with urinary incontinence at an older age.

*Benefit… I do not have to… change my pants when I get wet, right? This is the most basic. The second factor is personal dignity. It may be more troublesome when you are old; that is, in your later years, this symptom may worsen. If this symptom worsens, it is likely that one will be affected by self-esteem and be disliked by one’s family. Not being able to take care of yourself or cannot provide semi-self-care for yourself is very painful, right? Whether from one’s self, or the whole family life, it is an unnecessary trouble, right? Thus, from these dimensions, it is best to manage them well. If you exercise well in the early stages, it will be better in later stages. [P07]*

However, there were still some patients with urinary incontinence who doubted the effectiveness of pelvic floor management, and this can be a barrier to pelvic floor rehabilitation.

#### Interactions of interpersonal situations

##### Social influences

Participants discussed the impact of the sociocultural atmosphere on their decision to participate in pelvic floor rehabilitation behavioural programs and behaviour change. A few participants said that the Chinese society is currently in a collective unconscious stage on pelvic floor management, especially during pregnancy, as perceptions and awareness are lacking in most people. A few participants reported generational differences in their perceptions of pelvic floor management. Generational differences refer to differences in behaviour and thinking among people within a certain cycle owing to differences in family atmosphere, social experiences, etc. Compared with other generations (post-90 s and post-95 s), the older generation (post-80 s: refers to those born from 1 January 1980 to 31 December 1989) was less aware of pelvic floor management, and most of them considered urinary incontinence to be normal and rarely talked about urinary incontinence-related issues. We believe that there is a disconnect in the information to which younger and older generations are exposed.

*In fact, Chinese people and women do not exercise often, which is a common phenomenon. They also have less core strength. Then you let me go to exercise specifically for the pelvic floor muscles. Do people not even have the concept of exercising yet right? This is a problem. This is a collective unconsciousness. Now your post-90s may be a little better, but people like us are too lazy to move. This is a problem for the current generation. In fact, many of my colleagues were in the post-90s. Wow! They pay much attention to their body and perform Pilates and many other kinds of exercises. In fact, yoga is helpful for pelvic floor muscle recovery, so many of these things are actually the concept. However, we lack such concepts. [P07]*

In addition, the participants emphasised that because of the impetuous atmosphere of the society, everyone pursues short-term and fast-paced treatment programs; that is, they tend to prefer short-term and quick-acting treatment programs. However, pelvic floor management is a long-term process.

*Pelvic floor management is a very slow process because people are fast-paced. They want to see effects, and when these effects come, people are motivated to stick with it. It is like a fake advertisement for weight loss drugs, saying that you lose a few kilos a week. Moreover, this pelvic floor management may not be possible, and its strength is not so great; therefore, not many people who can accept it. [P17]*

Social support is an important driving factor for pelvic floor rehabilitation behaviours. Participants pointed out that different sources of social support were critical to promoting pelvic floor rehabilitation behaviours, mainly including the following:

1. Information and advice provided by the medical teams. Physicians' opinions and recommendations were considered drivers of behavioural changes in pelvic floor rehabilitation. Early mobilisation by influential clinicians promotes the participation in pelvic floor management.
2. Support from family members. Most participants reported that family support played a role in their behavioural changes. Family members' support, whether practical (such as a reminder) or emotional (such as understanding), was a driver, and its absence was a barrier.
3. Peer support. Peer support was an important factor in improving adherence. Peer support, such as the formation of offline or online groups for pelvic floor management where peers provide mutual supervision, can enhance social bonding and promote active participation by sharing practical advice and personal experiences. Participants reported valuing this support and described how its lack prevented them from making behavioural changes.
4. Network support from local communities and other groups. Group campaigns, carried out through organisations such as community and labour unions, are used to strengthen the publicity and promotion of pelvic floor rehabilitation for pregnant women, and provide support for promoting participants' adherence to pelvic floor management.

*Some communities have started to promote the two-child policy and other policies, whatever. I think the community should improve publicity on pelvic floor rehabilitation for women, mothers, etc. I think promoting this is a good idea. Because you know that the second child policy and breastfeeding have been promoted through the community. The policy of pelvic floor rehabilitation for women should also be synchronised in communities. [P20]*

#### Environment, resources, and decision-making processes

##### Memory, attention and decision processes

A few participants believed that they often had several daily tasks such as busy work schedules and demanding home responsibilities. Time limitation and forgetfulness, brought on by a variety of external factors, make it challenging for patients to focus on their own health and negatively affect their decision-making regarding pelvic floor rehabilitation behaviours.

*In addition to working, you also let me take time to exercise. I had many things to do, then I gave up after thinking about it and stopped exercising. This type of thing was ranked at the end of the list. You do not always lie down at home when you are on maternity leave. You have to do housework and take care of children and other members of the household, among other things, which take a lot of your time. Many people are not aware of pelvic floor management (laughter), including me. Because you have many things to do, you may think about other things. [P07]*

To overcome these external barriers, a few women expressed the importance of taking time away from home for exercise to focus on their personal health and optimise self-behaviour. Several participants suggested setting an alarm clock or clocking in as an effective way to remind women to exercise so as to focus their attention on pelvic floor self-management.

*There are several ways to stick to the exercise. If you regard it as a very important thing, can you also set up a kind of disciplined punch-in, right? It can also be done. [P03]*

##### Environmental context and resources

Participants reflected that they could obtain relevant information on pelvic floor management through various channels, among which are xiaohongshu, Tiktok, and other applications (APPs) widely used to retrieve information. A few respondents also indicated that they searched for relevant information on the Internet, such as in the Baidu Encyclopedia. However, some patients believed that the information available online are contradictory and unreliable, and they expressed concern about the authenticity of this information. Regarding information preference, the participants noted that they did not like to read complex and obscure content, as such information is often tedious and challenging to understand. Some participants pointed out that they preferred specific real cases, and they believed that through case sharing, they could feel the authenticity and reliability of the pelvic floor management effect, which could promote the implementation of pelvic floor rehabilitation behaviours.

*The hospital's (information) is more obscure because they are more summarised, just like textbooks. If you could conduct some interviews inside the hospital, for example, as was done today, with some real people, it might be more convincing. Like other official things, most people do not like to read textbooks, and those who do not like to read textbooks will (oops) fall asleep. However, if they are personal, that is, if you use living examples, we would be very willing to read such kind of information. [P17]*

#### Personal goal-setting and efforts towards behaviour change

##### Intentions

Intentions were considered both barriers and facilitators for patients to perform pelvic floor rehabilitation. Most participants expressed their intention to participate in pelvic floor management in the future. The participants stated that physical optimality was achieved by minimising the impact of urinary incontinence on daily activities through pelvic floor management. The participants believed that the severity of a patient's clinical condition may affect their decision and level of motivation to perform pelvic floor management. Patients who experienced less severe symptoms of urinary incontinence typically decide not to participate or have limited enthusiasm for pelvic floor management.

*The extent to which I adhere to pelvic floor management depends on my own situation. If I have severe urinary incontinence, I can manage my pelvic floor every day until I recover. But if I do not have severe symptoms, or if I do not have this condition, I may do very little, or I may not do any of this at all, and that is it. [P02]*

##### Goals

Compared to other daily imperatives, some participants did not consider pelvic floor management a priority for women and they did not associate urinary incontinence with unavoidable consequences.

##### Social / professional role and identity

In terms of responsibility for implementing pelvic floor rehabilitation behaviours, the participants emphasised on self-responsibility. The participants indicated that pelvic floor rehabilitation should be taken seriously by each individual to achieve self-management and change their disease status.

##### Behavioural regulation

Several participants reported using specific behaviour-changing strategies to enable them to perform long-term pelvic floor management. Most participants considered self-pelvic floor muscle exercises at certain times (such as before going to bed or when they performed specific actions) and formed their own exercise habits. These practical strategies can help change undesirable behaviours and increase the feasibility of performing long-term exercise.

*For example, if you perform pelvic floor muscle exercise as the pre-action of brushing your teeth, you must perform this exercise before brushing your teeth. [P19]*

##### Reinforcement

The participants emphasised the importance of appropriate incentives and material rewards to reinforce pelvic floor rehabilitation behaviours. This reinforcement has a positive impact on decision-making behaviour related to pelvic floor management.

#### Emotional influences on decision-making

##### Emotions

For some patients, urine leakage is embarrassing, resulting in negative psychological feelings, such as anxiety and nervousness, affecting self-esteem and creating an unnecessary emotional burden on daily life and family members. A few patients reported that a negative emotional state in daily life made it difficult to perform pelvic floor management, which is a barrier to pelvic floor rehabilitation behaviours. Some patients expressed positive views on the safety of pelvic floor muscle exercises as a promoting factor in pelvic floor rehabilitation.

#### Personal characteristics

This theme did not capture relevant data that matched the TDF domains. Personal characteristics include women's evaluations of their own and others' characteristics. Excellent personal characteristics such as independence, strong self-control, courage, and resilience, as well as a rational and calm attitude towards life, play a positive role in promoting pelvic floor management. However, the majority of participants reported that, while they recognised the importance of perseverance and willpower to adhere to pelvic floor management, they still had difficulty with persistence due to their laziness.

*Exercise requires persistence. If you persist, I think you can do so anytime anywhere if you realise that you have to do it. I think it is a habit that needs to be developed along with the will to persevere. [P14]*

## Discussion

### Main findings

To our knowledge, this is the first qualitative study using the TDF to explore the barriers and facilitators influencing participation in pelvic floor rehabilitation from the perspective of pregnant women with stress urinary incontinence. This study aimed to provide information on the development of pelvic floor rehabilitation intervention. Pelvic floor rehabilitation is essentially a self-management process, and adherence is key to effective pelvic floor management [14]. A previous study on promoting adherence to pelvic floor muscle exercises reported that the application of relevant behavioural change theories could promote the understanding of healthy behaviours, guide research, and facilitate the design and implementation of interventions to maximise behavioural adherence to pelvic floor rehabilitation [14]. Furthermore, studies have reported that adherence patterns established in the early stages promote long-term adherence [37]. Therefore, identifying the barriers and enablers of pelvic floor management during pregnancy not only helps to optimise the adherence to pelvic floor rehabilitation behaviours during pregnancy but also plays an important role in promoting postpartum and long-term pelvic floor rehabilitation.

Our findings suggest that lack of knowledge and skills are important barriers to pelvic floor rehabilitation behaviours in pregnant patients with stress urinary incontinence. Several patients have incorrect or very limited knowledge of when pelvic floor rehabilitation should begin and specific details of lifestyle management, and lack the skills and confidence to perform pelvic floor muscle exercises correctly. Previous studies have found that women face difficulties in obtaining information about pelvic floor management [38], and some are unable to contract the pelvic floor muscle correctly [39]. Lack of knowledge and skills may have adverse effects on other domains of TDF, such as beliefs about capabilities (the confidence of pregnant women with urinary incontinence in performing pelvic floor management behaviours). Studies have shown that women who acquire pelvic floor information are more likely to adhere to pelvic floor conservative treatments [14, 40]. It is worth noting that simple verbal instruction can help the majority of women (84%) who have difficulty understanding and performing pelvic floor muscle exercises to contract their pelvic floor muscles [41]. Therefore, to improve the knowledge and skill level of patients, healthcare professionals are encouraged to provide detailed health education to patients so that they can understand the anatomy and function of the pelvic floor muscles, master the ability to contract the muscles correctly, and increase their confidence in pelvic floor management.

Judgments about expected outcomes influenced the patients' pelvic floor rehabilitation behaviours. Previous investigations have found that minimally perceived benefit is a key barrier to adherence and that patients need visible or tangible effects to maintain adherence to pelvic floor rehabilitation [42]. However, pelvic floor rehabilitation behaviours take a long time to be effective. A few studies have pointed out that the benefits of lifestyle and behavioural strategies (mainly based on pelvic floor muscle exercises) on urinary incontinence are usually observed within 4–6 weeks [43]. Healthcare providers need to provide examples of women who have benefited from pelvic floor rehabilitation, and explain the benefits of pelvic floor rehabilitation behaviours (such as "the risk of urinary incontinence is reduced by 62% by the end of pregnancy") [42, 44], teach the patients the corresponding knowledge, and convince them of the effectiveness of pelvic floor muscle exercises in achieving long-term adherence and preventing adverse consequences.

Existing research supports our conclusion that social influence is a significant factor in behaviour modification [45]. Poor social and cultural atmosphere and ethos hinder pregnant women from participating in pelvic floor rehabilitation. On the one hand, there are generational differences in women's perceptions of pelvic floor management; compared to younger pregnant patients, older pregnant patients have less access to pelvic floor management information. Importantly, this study was conducted in the context of the Chinese government's December 2015 decision to lift the "one-child family" restriction and permit the "two-child family" policy. The change in government policy has increased women's willingness to have a second child, particularly among post-80 s women [46]. Unfortunately, however, the popularisation of pelvic floor rehabilitation knowledge is still a challenge in Chinese society. On the other hand, the impetuous social atmosphere has a detrimental impact on women's active participation in pelvic floor rehabilitation behaviours. In this context, health professionals should pay more attention to older pregnant women with stress urinary incontinence and impart relevant education in this population. Simultaneously, patients should be aware that pelvic floor rehabilitation is a slow-acting process and should avoid arrogance, develop good habits in their daily lives, and be psychologically prepared for long-term pelvic floor management.

Additionally, we discovered that social support from a variety of sources, including information and advice provided by the healthcare team, support from family members, peer support, and support from group organisations such as the local community, were all positive facilitators of pelvic floor rehabilitation behaviours in pregnant patients. Salmon et al. reported that pelvic floor muscle exercises without access to healthcare professionals' supervision might reduce women's ability to adopt and maintain training programmes [47]. Thus, the feedback provided by health professionals was also a condition for adherence to exercise [48]. According to research, the support of intimate relationships, such as spouses and family members, has a positive effect on women's behaviour change, which not only helps to promote women's healthy lifestyles but can also promote greater psychological resilience [45]. It should be noted that female relatives play an important role in women's participation in decision-making. Women may disregard advice from medical professionals if other female relatives believe it to be unimportant or irrelevant [47]. In addition, the mechanism by which peer support and support from group organisations, such as local communities, contribute to participation in pelvic floor rehabilitation involves the sharing of information and emotional exchange between support groups, making exercise a more enjoyable experience to enhance engagement and change health behaviours.

The environment and resources influence women's participation in pelvic floor management. Women often forget to perform pelvic floor rehabilitation exercises because of their busy work and family affairs, which consequently leads to low adherence. This result is consistent with a previous finding that the most common barrier to adherence is difficulty remembering to perform the exercise [19]. In addition, sustained cognitive attention is required to remember the exercise; however, cognitive attention to pelvic floor muscle exercises tends to decline over time [49]. To address this barrier, participants in our study suggested using strategies such as alarm clock reminders or punch-in, thus suggesting that patients may benefit from behaviour-changing strategies, such as punch-in or reminders. Furthermore, previous studies have reported that the adherence of patients participating in treatment can be improved using electronic reminders [50]. However, a randomised trial found that women who received additional video and reminder interventions showed no improvement in adherence compared with women who received traditional physical therapy alone [51]. Therefore, it is necessary to further explore the effectiveness of reminder strategies in improving adherence with pelvic floor rehabilitation behaviours.

When it comes to methods of accessing resources, patient-reported APPs and the internet are common ways to learn about prenatal care information. Many patients prefer to access treatment information from the Internet and APPs because they believe that this information is easy to obtain and has the characteristics of simplicity and vividness. There is evidence that mobile APPs can promote pregnancy self-management and improve health care services [52]. However, studies have reported that there are limited reliable sources of information related to electronic intervention and a lack of personal feedback and internal support, which can easily cause women to lose motivation for behavioural change [53, 54].

Prioritising other matters such as family obligations or work requirements is a significant barrier to pelvic floor management. We found that some women were accustomed to prioritising the needs of others over their own, and even putting pelvic floor management as the last priority in their lives. Similar results were found in the postpartum population [55]. Previous studies have also shown that a sense of responsibility and the need to take care of others are the biggest barriers for women to adhere to pelvic floor muscle exercises [56]. This is due to the fact that in Eastern countries, married women are expected to take on not only more housework while working long hours, but are also expected to play the roles of raising children and taking care of the elderly [57]. Despite the social changes that have taken place in the last few decades, when men have become increasingly involved in family care, women are still the primary caregivers, at least in the Chinese society.

Notably, the themes generated from the inductive analysis did not map to the "Optimism" domain of TDF. We found responses to the "Optimism" domain question ultimately matched the structural domain of "Beliefs about consequences" in the TDF. This result may be attributable to the structure and language of the questions we used. Although some TDF domains (e.g., "Beliefs about consequences" and "Optimism") are difficult to distinguish [58], previous research has noted that the optimism domain focuses on general disposition [22]. This study yielded themes of personal characteristics from the inductive analysis, but no data were found on whether women had optimistic or pessimistic characteristics in this theme. Although the inductive analysis generated themes that did not map to the "Optimism" domain of TDF, this does not necessarily mean that this domain is unimportant for adherence to pelvic floor rehabilitation behaviours. Previous research has demonstrated that patients with chronic illnesses who score higher on optimism have healthier behaviours [59]. More research is needed to explore the role of optimism in improving adherence in pregnant patients with urinary incontinence.

### Strengths and limitations

The major advantage of this study is that we used the validated TDF as a theoretical framework to comprehensively evaluate the barriers and drivers of pelvic floor rehabilitation behaviours in pregnant women with stress urinary incontinence. We provided a more detailed and theory-based understanding of the reasons for pelvic floor rehabilitation behaviours in pregnant women with stress urinary incontinence. Furthermore, theory-based interventions are more likely to result in behavioural changes that can improve health outcomes. Therefore, the findings of this study can be applied to the design of theory-based interventions using the TDF to guide the selection of behavioural change techniques and intervention components to facilitate the implementation of pelvic floor rehabilitation behaviours. Another strength of this study is that we conducted a thematic analysis before deductively mapping themes to the TDF to ensure that potential themes unrelated to the TDF were not lost. We found that the personal characteristics theme failed to map to the relevant TDF domains, which complements and expands the TDF theory. In addition, a semi-structured interview method was applied to allow patients to clarify their responses in depth. This enables a richer narrative and offers greater details on the barriers and facilitators surrounding pelvic floor rehabilitation behaviours in pregnant women with stress urinary incontinence.

Despite several strengths, this study has few limitations. First, the development of the interview schedule was based on TDF. However, if applied in a highly structured manner, participants may only respond with opinions that fit within the specified theoretical domains; this approach is rarely able to elicit ideas that are not relevant to the TDF [35]. Second, in this study, the specificity required in developing behaviour-changing interventions focused on the patients; it did not provide an in-depth description of factors influencing pelvic floor rehabilitation behaviours from the wider perspective of other relevant professionals such as nurses, doctors, and physiotherapists. Third, the interview was conducted in a hospital environment, which may interfere with the responses of the participants. The participants may have given a response that meets the social expectations to present the best of themselves, which may affect the data quality. Finally, this study was conducted at a single medical institution. Due to differences in medical level and social and cultural context, medical institutions in different regions may have different norms and practises for pelvic floor management. Consequently, the results should be generalised with caution. Future studies should be conducted to understand the experiences of pelvic floor management in populations from different countries and regions, and understand the differences in the barriers and enablers of pelvic floor rehabilitation behaviours among pregnant women in different levels of health care services and cultural contexts.

### Implications for research practice

The results of this study can be used to promote the design and implementation of interventions for pelvic floor rehabilitation. Based on the behaviour change wheel [60], the TDF representing barriers and enablers of pelvic floor rehabilitation behaviour can be systematically mapped to a wide range of intervention functions and behaviour change techniques. According to our findings, intervention strategies such as education, training, environmental restructuring (e.g., use of mass media such as radio and television to reinforce advocacy efforts and support the development of reminder apps), and modeling (e.g., mutual support in the form of mutual support groups) may improve adherence to pelvic floor rehabilitation behaviours in women.

## Conclusions

This study discusses the barriers and enablers of pelvic floor rehabilitation behaviours in pregnant women with stress urinary incontinence based on the TDF. We identified multiple barriers and facilitators, including knowledge, social, and environmental influences, which highlight the complexity of pelvic floor rehabilitation behaviours in pregnant patients with stress urinary incontinence. The implementation of pelvic floor rehabilitation behaviours requires a concerted effort from the whole health care system, including healthcare professionals, patients, society, and organisations. The findings confirm the need for multiple interventions to support pelvic floor management in pregnant women with stress urinary incontinence, focusing on enhancing knowledge and skills in pelvic floor care and using appropriate behaviour change techniques (such as prompts) to provide a supportive environment.

## Supplementary Information

**Additional file 1.** Consolidated criteria for reporting qualitative studies (COREQ) 32-item checklist.

**Additional file 2.** Semi-structured interview schedule.

## Data Availability

Redacted transcripts are available from the corresponding author upon reasonable request.

## Abbreviation

TDF: The Theoretical Domains Framework
