# Supplementary material for: Barriers and enablers of pelvic floor rehabilitation behaviours in pregnant women with stress urinary incontinence: a qualitative analysis using the theoretical domains framework
Source: BMC Pregnancy Childbirth. 2023 Apr 28;23:300. doi: 10.1186/s12884-023-05633-2 (PMC10148524; doi:10.1186/s12884-023-05633-2)
Supplement: Supplementary file 1 — Additional file 1. Consolidated criteria for reporting qualitative studies (COREQ) 32-item checklist. [file 12884_2023_5633_MOESM1_ESM.docx]

**Additional file 1.** Consolidated criteria for reporting qualitative studies (COREQ) 32-item checklist.

| **Item No.** | **Topic** | **Guide Questions/ Description** | **Reported on Page No.** |
| --- | --- | --- | --- |
| **Domain 1: research team and reflexivity** | | | |
| *Personal characteristics* | | | |
| 1 | Interviewer/facilitator | Which author/s conducted the interview or focus group? | 9 |
| 2 | Credentials | What were the researcher’s credentials? E.g. PhD, MD | 12 |
| 3 | Occupation | What was their occupation at the time of the study? | 12 |
| 4 | Gender | Was the researcher male or female? | All researchers were female / not reported in the manuscript |
| 5 | Experience and training | What experience or training did the researcher have? | 12 |
| *Relationship with participants* | | | |
| 6 | Relationship established | Was a relationship established prior to study commencement? | 12 |
| 7 | Participant knowledge of the interviewer | What did the participants know about the researcher? | 9 |
| 8 | Interviewer characteristics | What characteristics were reported about the interviewer/facilitator? | Participants were informed that the interviewer was a doctoral student, the research objectives, and that the study was conducted as part of the first author's PhD project / not reported in the manuscript |
| **Domain 2: study design** | | | |
| *Theoretical framework* | | | |
| 9 | Methodological orientation and Theory | What methodological orientation was stated to underpin the study? | 6, 10 |
| *Participant selection* | | | |
| 10 | Sampling | How were participants selected? | 7, 8 |
| 11 | Method of approach | How were participants approached? | 7 |
| 12 | Sample size | How many participants were in the study? | 12 |
| 13 | Non-participation | How many people refused to participate or dropped out? Reasons? | 12 |
| *Setting* | | | |
| 14 | Setting of data collection | Where was the data collected? | 9 |
| 15 | Presence of non-participants | Was anyone else present besides the participants and researchers? | 9 |
| 16 | Description of sample | What are the important characteristics of the sample? | 12, Table 3 |
| *Data collection* | | | |
| 17 | Interview guide | Were questions, prompts, guides provided by the authors? Was it pilot tested? | 9, Additional file 2 |
| 18 | Repeat interviews | Were repeat interviews carried out? If yes, how many? | 12 |
| 19 | Audio/visual recording | Did the research use audio or visual recording to collect the data? | 9 |
| 20 | Field notes | Were field notes made during and/or after the interview or focus group? | 9 |
| 21 | Duration | What was the duration of the interviews or focus group? | 12, Table3 |
| 22 | Data saturation | Was data saturation discussed? | 8, 12 |
| 23 | Transcripts returned | Were transcripts returned to participants for comment and/or correction? | 9 |
| **Domain 3: analysis and findings** | | | |
| *Data analysis* | | | |
| 24 | Number of data coders | How many data coders coded the data? | 11 |
| 25 | Description of the coding tree | Did authors provide a description of the coding tree? | Nil |
| 26 | Derivation of themes | Were themes identified in advance or derived from the data? | 10, 11 |
| 27 | Software | What software, if applicable, was used to manage the data? | 10 |
| 28 | Participant checking | Did participants provide feedback on the findings? | Nil |
| *Reporting* | | | |
| 29 | Quotations presented | Were participant quotations presented to illustrate the themes / findings? Was each quotation identified? | 14-24 |
| 30 | Data and findings consistent | Was there consistency between the data presented and the findings? | 13-24 |
| 31 | Clarity of major themes | Were major themes clearly presented in the findings? | Table 4, 13-24 |
| 32 | Clarity of minor themes | Is there a description of diverse cases or discussion of minor themes? | Table 4 |
