# Supplementary material for: Barriers and enablers of pelvic floor rehabilitation behaviours in pregnant women with stress urinary incontinence: a qualitative analysis using the theoretical domains framework
Source: BMC Pregnancy Childbirth. 2023 Apr 28;23:300. doi: 10.1186/s12884-023-05633-2 (PMC10148524; doi:10.1186/s12884-023-05633-2)
Supplement: Supplementary file 2 — Additional file 2. Semi-structured interview schedule. [file 12884_2023_5633_MOESM2_ESM.docx]

**Additional file 2.** Semi-structured interview schedule.

| **Open-ended questions** | |
| --- | --- |
| - How long have you been pregnant? | |
| - How have you felt during your pregnancy so far? | |
| - When did you first notice you were leaking urine? | |
| **TDF domain** | **Interview Questions** |
| Knowledge | - What do you know about urinary incontinence? (Prompts: classification, susceptible populations) - Do you know of any practices to prevent and treat urinary incontinence? For example, ways to reduce the risk of postpartum urinary incontinence. (Prompts: pelvic floor muscle exercise-the reasons, the time of onset, and duration; lifestyle interventions-weight loss, dietary changes, caffeine intake, fluid intake, smoking cessation, physical exercise, etc.) |
| Skills | - How do you usually control and manage urinary incontinence? What are the specific methods? - Do you know how to perform pelvic floor muscle exercise? - Do you think you have the skills to perform pelvic floor management correctly? (Prompts: Have you received any training on urinary incontinence management? What are your views on training? Do you need training?) |
| Memory, attention and decision processes | - Is performing pelvic floor management usually an easy or difficult decision to make? (Prompts: does it take time to think about, weigh up the pros and cons, etc.) - Have you decided to follow the pelvic floor management exactly as prescribed? Why/why not? - Have you decided not to do pelvic floor management in certain situations/times? (Prompt: Explain how you came to this decision. What factors would have helped your decision not to do pelvic floor management?) |
| Behavioral regulation | - Can you think of some ways or strategies to stick to pelvic floor management? If so, any examples? - If you wanted to start changing bad habits to effectively manage your urinary incontinence, what would you do? (Prompt: Do you actively stop all inappropriate exercise and food?) |
| Environmental context and resources | - Have there been problems in the past that prevented or interrupted pelvic floor management? Why was it interrupted? (Prompts: forgetfulness, too busy, support, time) - Are there sufficient information resources available? (Prompts: books, the internet) |
| Social influences | - Have you ever talked to someone about urinary incontinence before? Has anyone ever discussed the importance of pelvic floor management with you? (Prompts: Who? Health care professionals? A family member? A friend? What was discussed?) - Do you think the thoughts or practices of friends, family, colleagues, and healthcare professionals (family, work, or social environment) have influenced your decision to undertake pelvic floor management? (Prompt: How did it influence you? To what extent? Did they help or hinder? How have they supported you? reminding you? exercising with you?) |
| Social/professional role and identity | - As a patient with pregnancy urinary incontinence, do you think it is entirely your responsibility to manage your pelvic floor or should others be involved? Why? |
| Intentions | - Do you plan to stick to pelvic floor rehabilitation management in the future? Make pelvic floor management a part of your daily life. (Prompts: Why? Will this idea change over time?) - Are there any health behaviours that you are particularly keen to perform? - Are there any health behaviours that you are not keen to engage in? |
| Goals | - Compared to other priorities (things you have to do every day), how important is it for you to do pelvic floor management? (Prompts: on a scale of 0-10, 1 = lowest importance, 10 = highest importance; why?) - To what extent do you adhere to health management to promote pelvic floor rehabilitation? |
| Beliefs about consequences | - What do you think the effects of pelvic floor management (performing pelvic floor muscle exercises) during pregnancy will be for your body? (Prompts: physical or psychological effects. Potential benefits or disadvantages and why? Do you think the benefits outweigh the disadvantages?) - What do you think will happen if you do not manage your pelvic floor or do not manage it well? (Prompts: What health risks have you heard about? With family, with colleagues, with yourself, in the short and long term, e.g., going out, work, married life, etc.) |
| Optimism | - Do you think there is value in pelvic floor management? Why? |
| Beliefs about capabilities | - How confident are you in your ability to perform effective pelvic floor management? (Prompts: Manage yourself, ask for help) - How difficult or easy is it for you to perform pelvic floor rehabilitation behaviours? What parts of pelvic floor management are easy or difficult to accomplish? (Prompts: What problems or difficulties do you come across when performing management? Can you describe them in detail?) |
| Reinforcement | - What encourages or hinders you from pelvic floor management? - Do you know a patient who has suffered adverse consequences (e.g., prolonged postpartum urine leakage) due to a lack of active pelvic floor management? |
| Emotions | - How do you feel about urinary incontinence? (Prompts: Doesn't matter? Uncomfortable or embarrassed? Avoid dealing with it? Avoid consulting a healthcare professional on the subject?) Do your feelings or emotions affect your commitment to pelvic floor management? To what extent does it affect you? - What feelings or emotions did you have when you decided (not) to do and stick to pelvic floor management? (Prompts: positive or negative, e.g., did you feel happy, stressed, anxious, fearful?) |

One last question: Is there anything else you would like to say on this topic?
